# Islet Dysfunction in a Novel Transgenic Model of T Cell Insulitis

**DOI:** 10.3390/biom11040552

**Published:** 2021-04-09

**Authors:** Emily Esakov, Neha Nandedkar-Kulkarni, Ali G. Al-Dieri, Hannah Hafner, Brigid Gregg, Marcia F. McInerney

**Affiliations:** 1 Department of Medicinal and Biological Chemistry, College of Pharmacy and Pharmaceutical Sciences, University of Toledo, Toledo, OH 43614, USA; esakove@ccf.org (E.E.); nandedkarn@gmail.com (N.N.-K.); agaldieria2013@gmail.com (A.G.A.-D.); 2 Department of Pediatrics, Division of Pediatric Endocrinology, University of Michigan Medical School, Ann Arbor, MI 48109, USA; hhafner@med.umich.edu (H.H.); greggb@med.umich.edu (B.G.); 3 Department of Nutritional Sciences, School of Public Health, University of Michigan, Ann Arbor, MI 48109, USA; 4 Center for Diabetes and Endocrine Research, Department of Physiology and Pharmacology, College of Medicine and Life Sciences, University of Toledo, Toledo, OH 43614, USA

**Keywords:** pancreatic islets, insulitis, T cell, insulin receptor, glucose metabolism

## Abstract

The newly established CD3FLAG-mIR transgenic mouse model on a C57Bl/6 background has a FLAG tag on the mouse Insulin Receptor (mIR), specifically on T cells, as the FLAG-tagged mIR gene was engineered behind CD3 promoter and enhancer. The IR is a chemotactic molecule for insulin and the Flag-tagged mIR T cells in the BL/6-CD3FLAGmIR transgenic mice can migrate into the pancreas, as shown by immunofluorescent staining. While the transgenic mice do not become diabetic, there are phenotypic and metabolic changes in the islets. The transgenic islets become enlarged and disorganized by 15 weeks and those phenotypes continue out to 35 weeks of age. We examined the islets by RT-PCR for cell markers, ER stress markers, beta cell proliferation markers, and cytokines, as well as measuring serum insulin and insulin content in the pancreas at 15, 25, and 35 weeks of age. In transgenic mice, insulin in serum was increased at 15 weeks of age and glucose intolerance developed by 25 weeks of age. Passage of transgenic spleen cells into C57Bl/6 RAG^−^/^−^ mice resulted in enlarged and disorganized islets with T infiltration by 4 to 5 weeks post-transfer, replicating the transgenic mouse studies. Therefore, migration of non-antigen-specific T cells into islets has ramifications for islet organization and function.

## 1. Introduction

Insulin receptor (IR) expression on the T cell surface has a major contribution to T cell function and adaptive immunity [1]. Resting or naïve T cells have low levels of IR expression on their surface and are insensitive to insulin [2]. However, IR surface expression emerges upon T cell activation [3,4]. Insulin binds to the IR on stimulated T cells and initiates glucose uptake, glucose and lipid metabolism, protein synthesis, and amino acid transport. Insulin-IR interactions help T cells maintain an activated state, enhance cytotoxic function, and support growth and differentiation [3]. Interestingly, IR on the T cell surface also serves a chemotactic function. Therefore, IR^+^ T cells can move towards a source of insulin [5,6]. Chinese hamster ovary (CHO) cells normally have a low density of IR on their surface. However, overexpression of human IR via transfection on CHO cells allows for chemotaxis towards a source of insulin [7]. Berman et al. reported that in vitro activated IR^+^CD3^+^ T cells show a chemotactic response towards porcine insulin while resting T cell with low level of IR expression did not migrate significantly [5]. This study suggests that activated IR^+^ T cells can infiltrate, in vivo, into the insulin-producing pancreatic islets [5].

In type 1 diabetes (T1D), autoreactive immune cells infiltrate into the pancreatic islets resulting in loss of insulin-producing beta cells and ultimately leading to hyperglycemia [8]. Pancreatic tissue from humans with T1D has cellular lymphocytic infiltrate in the islets, termed as insulitis, which is thought to represent an immune response [9]. In T1D autoimmunity, T cells are major contributors to beta cell destruction [10,11,12,13,14,15,16]. A CD4^+^ or CD8^+^ T cell response depends on antigen presentation by the MHC molecule on the cell surface. However, recent findings suggest that antigen is not necessary for T cell trafficking into the islet, but rather depends on T cell chemokine receptor signaling and a chemokine gradient secreted by pancreatic beta cells [17].

In healthy individuals, pancreatic islets do not express MHC class II molecules and weakly express MHC class I molecules [18]. While MHC class I expression is increased on islets in both NOD mice and humans with T1D [19,20], MHC class II expression has not been observed in NOD mice [20]. In addition, transgenic expression of MHC class II on islets does not lead to hyperglycemia [21]. Hamilton-Williams et al. [22] showed that T cells will traffic into the pancreas in NOD mice genetically engineered not to express MHC class I on the islet cell surface. Therefore, insulitis occurred in these mice without T cell-MHC/antigen-specific interactions, and the insulitis was benign and did not progress to beta cell destruction.

Transgenic FVB mice expressing FLAG-tagged IR specifically on T cells were engineering by placing the FLAG-tagged mouse IR gene behind the CD3 promoter and enhancer [23]. Transgenic FVB mice showed evidence of non-antigen-specific insulitis by H&E staining as early as six weeks of age [23]. Newly established transgenic C57BL/6 mice expressing FLAG-tagged IR specifically on T cells were engineered the same way as the FVB transgenic mice [23,24]. Purified CD3^+^ T cells from the transgenic BL/6-CD3FLAGmIR mice significantly chemotaxed to insulin as well as to islet cells in vitro as compared to C57BL/6 controls [24]. BL/6-CD3FLAGmIR transgenic mice, an in vivo model of IR^+^ T cell non-antigen-specific migration to the pancreas, showed evidence of insulitis into the islets by H&E, immunofluorescence (IF), immunohistochemistry (IHC) and IF double staining for insulin, and the FLAG tag on the T cells [24]. By RT-PCR analysis of isolated islets CD8 T cells were significantly expressed by 25 weeks of age and 35 weeks of age. However, the insulitis was benign, and the animals did not progress to hyperglycemia out to 45 weeks of age [24]. Therefore, morphological and metabolic changes in the BL/6-CD3FLAGmIR transgenic mice were studied to understand the effect on insulin-producing beta cells, and islet cells in general, of non-antigen-specific IR^+^ T cell migration into the pancreas.

## 2. Materials and Methods

### 2.1. Mice

Generation of BL/6-CD3FLAGmIR transgenic mice was described previously [24]. Age-matched C57BL/6 (BL/6) mice were used for all the experiments as controls. These mice were housed at the University of Toledo (Toledo, OH), under specific-pathogen-free conditions, in the Department of Laboratory Animal Resources. All animal experiments were performed according to approved guidelines of the National Institutes of Health and protocols approved by the Institutional Animal Care and Use Committee (protocol #105461-14, approved 21 September 2018). Due to the delay (5 to 10 weeks) of phenotype, in the transgenic female mice, the results being reported were obtained with male BL/6-CD3FLAGmIR mice and age-matched BL/6 male controls.

### 2.2. Islet Isolation

Islets were obtained by digesting the pancreas with 5 mL of ice-cold HANK’s balanced salt solution (HBSS, Life Technologies, Grand Island, NY, USA) containing 1 mg/mL Collagenase P (Sigma, St. Louis, MO, USA) at 37 °C for 12 min [25]. Digestion was blocked by the addition of 10 mL ice cold HBSS solution containing 10% fetal bovine serum (FBS, Atlanta Biologicals, Flowery Branch, GA, USA). The cell suspension was mixed and centrifuged at 1000 rpm for 2 min. The washing step was repeated twice, and the cell suspension was passed through a 500 µm filter in order to remove undigested tissue. The filtered cell suspension was then passed through a 70 µm filter. As islets remain on the 70 µm filter, the filter was washed thoroughly upside down using 15 mL complete media containing RPMI 1640 complete medium supplemented with Glutamine (Hyclone, South Logan, UT, USA), 1% penicillin-streptomycin (Mediatech Inc., Manassas, VA, USA), 10% fetal bovine serum (Atlanta Biologicals, Flowery Branch, GA, USA), 1% Sodium pyruvate (Life Technology, Grand Island, NY, USA), 1% non-essential amino acids (Mediatech Inc., Herndon, VA, USA), 0.001% 2-mercaptoethanol (Life Technology, Grand Island, NY, USA) and 5 mM D-glucose at 37 °C. Islets were handpicked under the microscope and incubated overnight in complete media at 37 °C or used immediately depending upon the experiment. Routinely, 50–150 islets were isolated per mouse.

### 2.3. mRNA Analysis by Quantitative Real Time PCR (qRT-PCR)

Islets or entire pancreatic tissue was obtained from 15-, 25- and 35-week-old male BL/6 and BL/6-CD3FLAGmIR mice as mentioned above. Islets were handpicked under the microscope, and whole pancreas was crushed using a glass homogenizer before being lysed and total mRNA purified using RNeasy mini kits (Qiagen, Hilden, Germany). cDNA was synthesized using M-MLV Reverse Transcriptase (Thermo Fisher Scientific, Pittsburg, PA, USA) and quantitated via the BioSpec-nano (Shimadzu Biotech, Columbia, MD, USA). The optimized reaction conditions for qRT-PCR analysis are mentioned in Table 1. Mouse Glyceraldehyde-3 phosphate dehydrogenase (GAPDH) or beta-globin was used (noted in figure legends) to normalize the relative amount of mRNA using the delta Ct method for quantitative analysis. Primers for qRT-PCR are reported in Table 2. All the primers were obtained from Integrated DNA Technology (San Jose, CA, USA). Experimental protocol used for qRT-PCR study in the CFX96 system Thermocycler system (Bio-Rad, Hercules, CA, USA) is mentioned in Table 3. The use of qPCR to investigate the infiltration of expression markers in islets has been previously described [26,27].

### 2.4. Intraventricular Perfusion

BL/6 and BL/6-CD3FLAGmIR mice were anaesthetized by 100 µL intraperitoneal injection of sterile Ketamine (VetOne, Boise, ID, USA)/Xylazine (AnaSed inject, Greeley, CO, USA) solution (Ketamine 10 mg/kg body weight of mice, Xylazine 12.5 mg/kg). Once the animal reached a surgical plane of anesthesia determined by toe-pinch response method, the incision was made through the abdomen and was the length of the diaphragm. Further cut was made through the thoracic cavity and heart was exposed for drainage of blood and fluids by proper clamping of tissues around the heart. Phosphate buffer saline (PBS) (Thermo Fisher Scientific, Pittsburg, PA, USA) was perfused through the heart first after making an incision to the animal’s right atrium. This helps to remove the blood from blood vessels. Later 4% paraformaldehyde (Thermo Fisher Scientific, Pittsburg, PA, USA) solution was perfused through heart for fixing the animal tissue. Tissues were collected from the intraventricular perfused animal.

### 2.5. Immunofluorescence Staining (IF)

Pancreas tissue was harvested from BL/6 and BL/6-CD3FLAGmIR mice and fixed in 10% *w*/*v* formalin (Thermo Fisher Scientific, Pittsburg, PA, USA) at 4 °C overnight. Slides were de-paraffinized and rehydrated before antigen retrieval, by microwaving in 10 mM citrate buffer pH 6.0. Tissue sections were marked with the hydrophobic barrier. Section was covered with 100 µL blocking buffer, 1% Bovine Serum Albumin in PBS (BSA, Sigma, St. Louis, MO, USA). Sections were stained overnight at 4 °C with anti-FLAG FITC (1:100 Sigma, St. Louis, MO, USA), anti-CD3 APC (1:100, eBioscience, San Diego, CA, USA), guinea pig anti-insulin (1:1500, Sigma, St. Louis, MO, USA), washed and stained with secondary anti-guinea pig IgG Texas Red (1:500, Invitrogen/ Thermo Fisher Scientific Pittsburgh, PA, USA) for 1–2 h at RT. Primary and secondary antibody solution was prepared in the blocking buffer. All staining was performed in humidity chamber to avoid tissue drying and solution evaporation. After washing, sections were mounted with 4′,6-diamidino-2-phenylindole (DAPI)-Fluoromount G (Sigma, St. Louis, MO, USA) following dehydration and imaged on a Nikon TS fluorescent microscope (Nikon Instruments, Melville, NY, USA).

### 2.6. Histological Microscopy

Pancreas tissue was harvested from 15-, 25-, and 35-week-old male BL/6 and BL/6-CD3FLAGmIR mice after intraventricular perfusion and fixed in 10% *w*/*v* formalin at 4 °C overnight. Sections were labelled for Hematoxylin and Eosin (H&E, Thermo Fisher Scientific, Pittsburg, PA, USA) after de-paraffinization and rehydration of tissue slides. After washing, sections were mounted with Fluoromount G mounting medium (Sigma, St. Louis, MO, USA) and imaged on a Cytation 5 Tissue Imager (BioRad, Hercules, CA, USA) or an Olympus VS120 slide scanner (Olympus, Center Valley, PA, USA) on brightfield setting.

### 2.7. Terminal Deoxynucleotidyl Transferase dUTP Nick End Labeling (TUNEL) Assay

Pancreas tissue was harvested from 15 and 25-week-old BL/6 and BL/6-CD3FLAGmIR mice after intraventricular perfusion and fixed in 10% *w*/*v* formalin at 4 °C overnight. TUNEL staining was performed according to manufacturer’s protocol (TACS 2TdT-3,3’ diamino benzidine tetrachloride (DAB) In Situ Apoptosis Detection Kit, Trevigen, Gaithersburg, MD, USA). All the components required for TUNEL assay were included in TACS 2TdT-DAB In Situ Apoptosis Detection Kit (Trevigen, Gaithersburg, MD, USA). The Slides were prepared and processed as described above in IF staining. Tissue section was marked with the hydrophobic barrier. Tissue section was covered with 50 µL Proteinase K solution (1:50 in Deionozed (DI) water) for 30 min. Tissue slide was washed with DI water twice for 2 min and immediately immersed in a quenching solution (5 mL of 30% hydrogen peroxide in 45 mL methanol) for 5 min. Washing step was repeated on the tissue slide with PBS for 1 min and later immersed in 1× TdT labelling buffer for 5 min. The tissue section was covered with 50 µL of labelling reaction mix (1 µL of TdT dNTP mix, 1 µL of TdT enzyme, 1 µL of 50× manganese cation stock and 50 µL of 1× TdT labeling buffer) and incubated for 60 min at 37 °C in a humidity chamber to avoid evaporation. The tissue slide was immersed in 1× stop buffer for 5 min and washed with DI water twice, 5 min each. 50 µL of strep-Horseradish Peroxidase (HRP) solution (1:50 in PBS) was added on the tissue section and incubated for 10 min at 37 °C in a humidity chamber. Strep-HRP treated tissue slide was washed with PBS twice for 2 min and immersed in DAB solution (50 mL PBS, 250 µL DAB, 50 µL DAB enhancer and 50 µL of 30% hydrogen peroxide) for 10 min. Later, tissue slide was washed with DI water twice for 2 min. Tissue slide was immersed in 1% Methyl green for 5 min in order to stain the nuclei. After washing, the tissue section was mounted with glass coverslip using Fluoromount-G mounting medium (Southern Biotech, Birmingham, AL, USA) following dehydration and imaged on an Olympus VS120 slide scanner (Olympus, Center Valley, PA, USA). ImageJ software was used to determine the TUNEL^+^ cells per islet.

### 2.8. Ki67 Proliferation Assay

Pancreas tissue was harvested from 15-week-old male BL/6 and BL/6-CD3FLAGmIR mice after intraventricular perfusion and fixed in 10% *w*/*v* formalin at 4 °C overnight. The Slides were prepared and processed as described above in IF staining. Tissue section was marked with the hydrophobic barrier. Tissue Section was covered with 100 µL blocking buffer (9 mL FBS, 9 mL of 10× Roche blocking buffer (Roche, Basel, Switzerland) and 27 mL of autoclaved maleate buffer (11.6 gm of Maleic acid (100 mM), 8.8 gm of sodium chloride (150 mM) and 800 mL of DI water)) for 30 min at RT. Tissue section was covered with 100 µL of primary antibody solution containing Rabbit Ki67 monoclonal antibody (1:200, Cell Signaling, Danvers, MA, USA), Guinea pig insulin polyclonal antibody (1:500, Dako, Santa Clara, CA, USA) and incubated overnight at 4 °C. Sample slide was washed with tris buffered saline (TBS, Sigma, St. Louis, MO, USA) with tween 20 (TBST Sigma, St. Louis, MO, USA) solution (50 mL of 20× TBS, 1 mL of tween 20 and diluted to 1000 mL with DI water) three times for 5 min with shaking. The section was covered with 100 µL of secondary antibody containing Cy3 conjugated Donkey anti-rabbit IgG (1:250, Jackson Immunoresearch, West Grove, PA, USA) and FITC conjugated Donkey anti-guinea pig IgG (1:250, Jackson Immunoresearch, West Grove, PA, USA) and incubated for 1 h at RT. Primary and secondary antibody solution was prepared in the blocking buffer. Sample slide was washed three times with TBST for 5 min with shaking. Nuclei were stained with 300 µL of 300 nM DAPI solution (1.327 µL of DAPI in 50 mL of PBS, Molecular Probe, Eugene, OR, USA) for 5 min. All staining was performed in humidity chamber to avoid tissue drying and solution evaporation. Slide was rinsed three times with PBS for 5 min each. After washing, section was mounted with glass coverslip using Fluoromount-G mounting medium (SouthernBiotech, Birmingham, AL, USA) and imaged on a Nikon TS fluorescent microscope (Nikon Instruments, Melville, NY, USA). ImageJ software was used to determine the number of Ki67^+^ cells and insulin positive beta cells in islets. Percentage of Ki67 was based on the number of Ki67^+^ cells per 500 beta cells.

### 2.9. Peripheral Blood Serum Insulin Level Analysis

Blood was collected from BL/6 and BL/6-CD3FLAGmIR mice from the tail vein using microvette capillary blood 300 EDTA (Kent Scientific, Torrington, CT, USA). Samples were centrifuged at 15,000 rpm for 15 min after keeping the samples at RT for 1 h. Supernatant serum was collected and used to perform mouse insulin ELISA (Alpco ultra-sensitive mouse insulin ELISA kit, Alpco, Salem, NH, USA).

### 2.10. Total Pancreatic Insulin Content ELISA Analysis

The pancreas was homogenized in ice cold acid ethanol (Thermo Fisher Scientific, Pittsburgh, PA, USA) (1 mL of 2N HCl in 110 mL of 95% ethanol). The homogenized solution was shaken at 4 °C for 72 h. After the incubation, the pancreatic extracts were centrifuged at 4 °C at 1000× *g* for 30 min. The clear supernatant was analyzed for the total insulin content using an ultra-sensitive mouse insulin ELISA kit (Alpco, Salem, NH, USA). The level of insulin content was normalized to the total protein content, which was determined using a BCA protein assay kit (Thermo Fisher Scientific, Pittsburg, PA, USA).

### 2.11. Intraperitoneal Glucose Tolerance Testing (IPGTT)

Ear-tagged 15-, 25-, and 35-week-old BL/6 and BL/6-CD3FLAGmIR mice were fasted 14–17 h in alpha dry bedding cages and baseline blood glucose was monitored by tail vein (Accu Chek Aviva Plus-Roche, Basel, Switzerland or Alpha Trak 2- Zoetis, Parsippany-Troy Hills, NJ, USA). Animals were weighed and a bolus of 50% dextrose (Hospira, Lake Forest, IL, USA) was administered through IP injection (4× bodyweight). Animals were left in a quiet atmosphere and blood glucose was monitored every 30 min for 3 h post-injection.

### 2.12. Glucose Stimulated Insulin Secretion Studies In Vitro

Islet isolation was performed as mentioned above in the islet isolation section. Islets were handpicked under the microscope and incubated overnight in complete media at 37 °C. The following day, islets were preincubated in Krebs-Ringer solution containing 2 mM glucose for 1 h at 37 °C. Later, 10 islets were seeded on the membrane of an insert (Millipore Sigma, Burlington, MA, USA) and placed in 24 well plates in triplicate. Islets were treated with Krebs- Ringer solution containing low D-glucose solution (2.5 mM) for 1 h. Buffer was collected and the inserts with islets were transferred into high glucose solution (22 mM) for 30 min. Buffer was again collected and the same inserts were transferred to 30 mM KCl solution for 15 min. After 15 min, supernatants were collected. Islets were treated with DNA lysis buffer (Cell Signaling, Danvers, MA, USA), sonicated, and then total DNA was measured by Qbit Fluorimeter (Life Technologies, Carlsbad, CA, USA). Insulin release was assayed on the collected solution using an ultra-sensitive mouse insulin ELISA kit (Alpco, Salem, NH, USA). Insulin release was normalized to islet DNA content.

### 2.13. Transgenic Splenocyte Adoptive Transfer into RAG^−/−^ Recipients

For adoptive transfer screening, IPGTT testing was completed on donor 25-week-old male BL/6 and BL/6-CD3FLAGmIR mice to analyze the metabolic effects of glucose challenge. The 3 BL/6-CD3FLAGmIR mice that had the poorest glucose clearance after glucose challenge and 3 BL/6 mice were used for adoptive transfer. Upon donor sacrifice, pancreatic tissue was taken and processed for histology. IF staining was completed to observe insulin staining intensity and islet size and *n* = 10 islets per each of the 3 mice per group were analyzed to additional confirm the results of the IPGT testing. Twenty million splenocytes from donors were harvested and after ACK lysis were adoptively transferred through retro-orbital injection into C57BL/6 RAG^−/−^ recipient mice. Four mice received BL/6-CD3FLAGmIR mice splenocytes, four mice received BL/6 splenocytes, and three mice received vehicle PBS. Random blood glucose was monitored, and recipients were sacrificed at pre-determined time points (4–5 weeks post-transfer). Recipient organs were collected for histology studies. Blood serum was collected for determining insulin levels.

### 2.14. Statistics

All data are presented as mean ± SEM and were analyzed using the unpaired Student’s *t* test or Mann–Whitney test or Welch’s *t* test or one-way ANOVA followed by Tukey’s multiple comparison test or two-way ANOVA followed by Tukey’s multiple comparison test depending upon the experimental design and variables in the experiment. Unpaired student *t* test was used for qRT-PCR, percentage of Ki67 proliferation, and glucose tolerance test data analysis of the age-matched BL/6 and BL/6-CD3FLAGmIR mice. One way ANOVA was used for blood glucose level, and TUNEL^+^ apoptosis marker data analysis of the BL/6 and BL/6-CD3FLAGmIR mice. Two-way ANOVA was used for the serum insulin level and total insulin content data analysis of the BL/6 and BL/6-CD3FLAGmIR mice. Mann–Whitney test was used for the islet size and beta cell nuclei data analysis of age-matched BL/6 and BL/6-CD3FLAGmIR mice. Welch’s *t* test was used for GSIS data analysis of age-matched BL/6 and BL/6-CD3FLAGmIR mice. *P* ≤ 0.05 was considered significant. All tests were performed using GraphPad Prism 5.04 software (GraphPad Software Inc., La Jolla, CA, USA).

## 3. Results

### 3.1. Characterization of IR^+^ T Cells Infiltrating into the Pancreatic Islet

**Figure 1:**
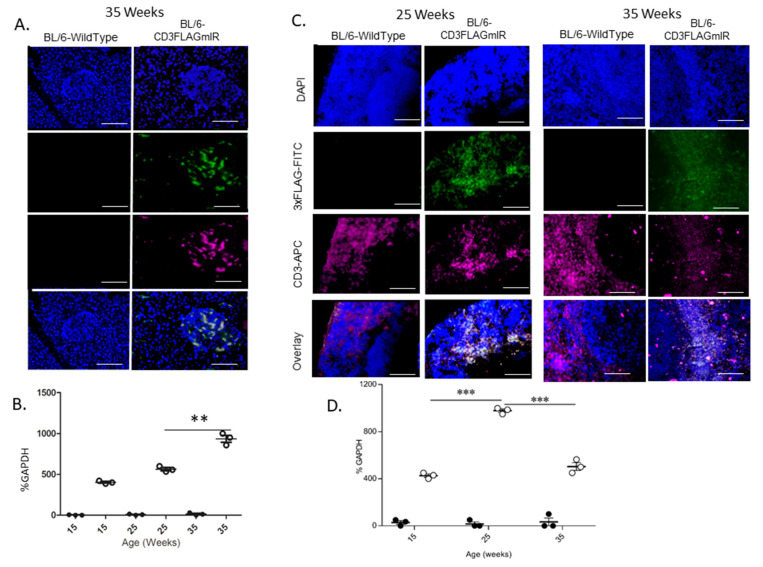
Phenotypic study of FLAG mouse Insulin Receptor (mIR) expression on pancreatic and splenic tissue of BL/6 and BL/6-CD3FLAGmIR male mice. Immunofluorescence (IF) staining for CD3^+^ T cells and FLAGmIR transgene expression. Pancreatic at 35 weeks of age (**A**) and splenic at 25 and 35 weeks of age (**C**) paraffin tissue sections were stained using anti-FLAG FITC labeled antibody (green), anti-CD3 APC labeled antibody (magenta), and DAPI-UV stain for nuclei (blue) and imaged at 20x. Scale bar: 300 µM. qPCR analysis of transgene 3xFLAGmIR in pancreatic (**B**) and splenic (**D**) tissue at 15, 25, and 35 weeks of age in BL/6 (black circles) and BL/6-CD3FLAGmIR transgenic (white circles) male mice. (*n* = 3 mice per group). One Way ANOVA, ** *p* < 0.01, *** *p* < 0.001.

Previously, we demonstrated that IR^+^ T cells infiltrate into the pancreatic islet of BL/6-CD3FLAGmIR mice and caused insulitis at 15 and 25 weeks of age by IF [24]. In Figure 1A, CD3^+^ IR^+^ T cells identified by the FLAG tag on the mIR are shown in the islet at 35 weeks of age. This figure is shown to demonstrate that the insulitis was not transient and is stable over time. Additionally, qRT-PCR analysis of the CD3FLAGmIR transgene is significantly increased by 35 weeks of age in the pancreas (Figure 1B). RT-PCR analysis of islets showed that CD3^+^ FLAG-Tagged mIR^+^ T cells and CD8^+^ T cells were significantly increased by 35 weeks of age [24]. Labeling CD3 and the FLAG tag on the transgenic T cells by IF was reported in the spleen and the thymus at 15 weeks of age [24]. Here we present data for 25 weeks and 35 weeks in the spleen by IF to show that FLAG-tagged IR^+^ T cells are represented in the spleen over time (Figure 1C). Interestingly, as pancreatic CD3FLAGmIR transgene increased at 35 weeks of age, the splenic transgene is significantly decreased compared to 25 weeks of age, indicating a possible shift of the transgenic splenic IR^+^ T cells to the pancreas (Figure 1B compared to Figure 1D).

**Figure 2:**
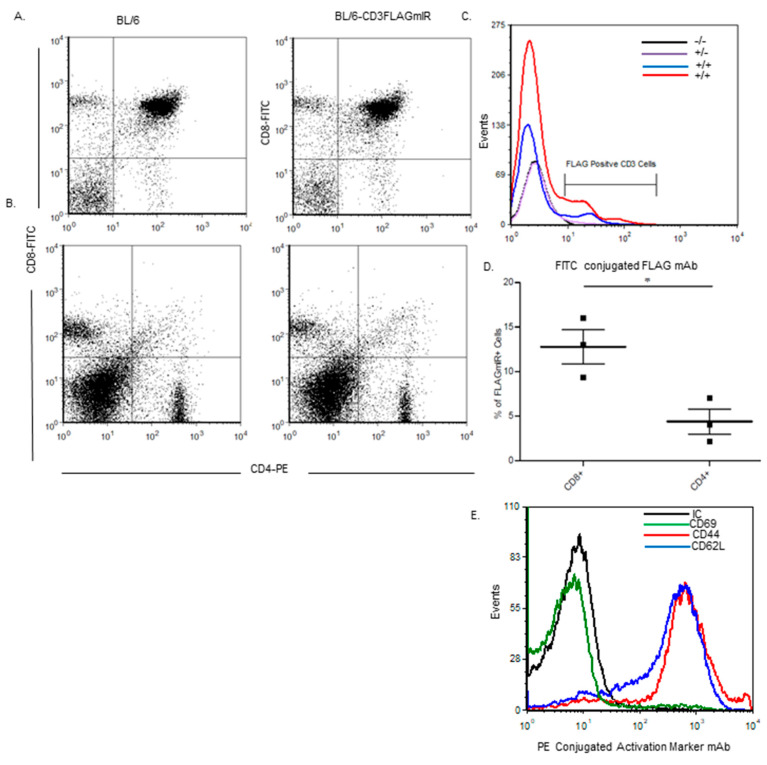
Study of CD4, CD8, FLAG expression, and activation markers on splenocytes or thymocytes of 25-week-old BL/6 and Bl/6-CD3FLAGmIR mice. (**A**) Expression comparison of thymic cell subpopulations of BL/6 and Bl/6-CD3FLAGmIR mice by flow cytometry, stained with anti-CD4 FITC, anti-CD8 PE, and anti-CD3-APC antibodies. The dot plots shown are gated on the CD3-APC-positive cell population from whole thymus (**B**) Expression comparison of splenic cell subpopulations of BL/6 and BL/6-CD3FLAGmIR mice by flow cytometry, stained with anti-CD4 FITC, anti-CD8 PE, and anti-CD3-APC antibodies. The dot plots shown are gated on the CD3-APC-positive cell population from whole spleen. (**C**) Splenocytes stained for expression of FLAGmIR transgene and gated on the CD3-APC-positive population. The black line (control) overlaps the violet line except at the end. (**D**) Percentage of FLAG^+^CD8^+^ versus FLAG^+^CD4^+^ splenocytes from BL/6-CD3FLAGmIR mice at 25 weeks of age. (**E**) Adhesion and Activation Marker analysis of transgene FLAGmIR^+^ splenocytes stained with either anti-CD44 PE, anti-CD62L PE, or anti-CD69PE and gated on the CD3^+^, FLAGmIR^+^-positive cell population. FLAGmIR^+^T cells from Bl/6-CD3FLAGmIR mice are CD44^+^ and CD62L^+^. These data are representative of 3 separate experiments. Unpaired student *t* test: * *p* ≤ 0.05.

Flow cytometry analysis was performed on splenocytes and thymocytes to investigate variations in CD3, CD4, and CD8 T cell populations between wildtype and transgenic mice. There were no significant differences in proportions of subsets of T cells, demonstrating that the transgene did not affect T cell development (Figure 2A,B). Transgenic mice also had differential expression of the CD3FLAGmIR transgene dependent on their genotype, with heterozygous mice expressing less than homozygous mice (Figure 2C). Even homozygous mice had different levels of expression of IR^+^ T cells, therefore results from two homozygous mice are shown in Figure 2C. Upon further investigation, the CD3FLAGmIR transgene was more highly expressed on the CD8 T cell subtype at 25 weeks of age (Figure 2D) and furthermore, the IR^+^ CD3^+^ T cells are CD62L^+^ CD44^+^, which are central memory markers (Figure 2E).

**Figure 3:**
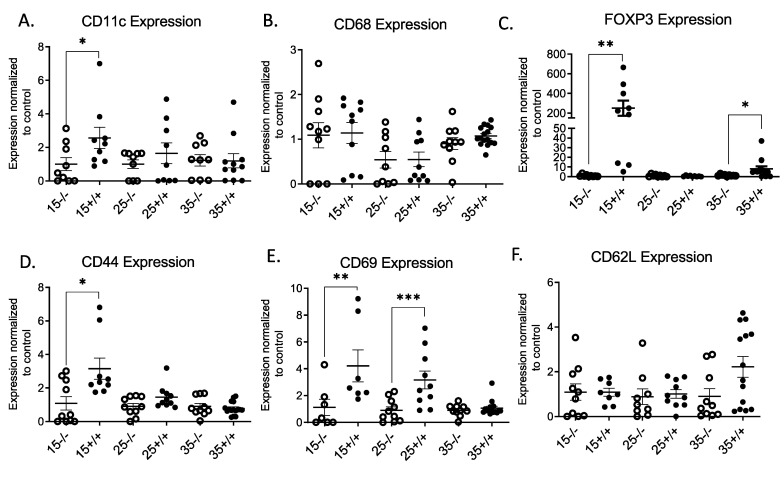
qRT-PCR analysis of BL/6 and BL/6-CD3FLAGmIR mice islet mRNA expression of CD11c, CD68, FOXP3, CD44, CD69, and CD62L. CD11c (**A**), CD68 (**B**), FOXP3 (**C**), CD44 (**D**), CD69 (**E**), and CD62L (**F**) islet mRNA expression at 15 (*n* = 3 mice with 3 replicates), 25 (*n* = 3 mice with 3 replicates) and 35 weeks (*n* = 4 mice with 3 replicates) of age. ^−/−^ indicates BL/6 mice and ^+/+^ indicates BL/6-CD3FLAGmIR mice. Both BL/6 and BL/6-CD3FLAGmIR results are compared to the control, beta-globin, a housekeeping gene. Unpaired student *t* test: * *p* ≤ 0.05, ** *p* ≤ 0.01, *** *p* ≤ 0.001.

A significant expression of CD11c by qPCR with no significant change in the CD68 level was observed in the BL/6-CD3FLAGmIR mouse islets at 15 weeks of age (Figure 3A,B). In BL/6-CD3FLAG mouse islets, FOXP3^+^ T regulatory cell mRNA expression was higher compared to BL/6 islets at 15 and 35 weeks of age (Figure 3C). CD44 and CD69 mRNA expression was increased in the 15-week-old BL/6-CD3FLAGmIR mouse islets (Figure 3D,E). Increase in the CD44 and CD69 levels indicates the presence of activated T cells in the islets of transgenic mice. CD69 expression was also significantly higher in the 25-week-old BL/6-CD3FLAGmIR mice islets (Figure 3E). CD62L mRNA expression did not change in the BL/6-CD3FLAGmIR mouse islets (Figure 3F).

**Figure 4:**
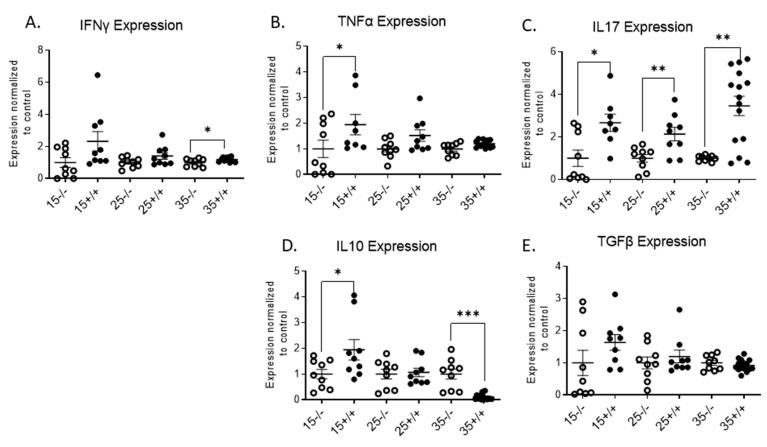
qRT-PCR analysis of BL/6 and BL/6-CD3FLAGmIR mice islet mRNA expression of IFNγ, TNFα, IL-17, IL-10, and TGFβ. IFNγ (**A**), TNFα (**B**), IL-17 (**C**), IL-10 (**D**), and TGFβ (**E**) islet mRNA expression at 15 weeks (*n* = 3 mice with 3 replicates), 25 weeks (*n* = 3 mice with 3 replicates) and 35 weeks (*n* = 4 mice with 3 replicates) of age. ^−/−^ indicates BL/6 mice and ^+/+^ indicates BL/6-CD3FLAGmIR mice. Both BL/6 and BL/6-CD3FLAGmIR results are compared to the control, beta-globin, a housekeeping gene. Unpaired student *t* test: * *p* ≤ 0.05, ** *p* ≤ 0.01, *** *p* < 0.001.

In order to confirm the presence of activated T cell in the transgenic mice islets, cytokine expression in the islets was evaluated. IFNγ was significantly increased at 35 weeks of age in the BL/6-CD3FLAGmIR mouse islets compared to BL/6 mice (Figure 4A). TNFα was significantly increased compared to BL/6 only at 15 weeks of age (Figure 4B). In addition, IL-17 expression was increased in the BL/6-CD3FLAGmIR mouse islets at all age points (Figure 4C). A significantly increased IL-10 expression was observed in the 15-week-old BL/6-CD3FLAGmIR mouse islets but was significantly lower in the transgenic mouse islets at 35 weeks of age compared to the 15- and 25-week-old transgenic mice (Figure 4D). However, we did not observe a significant change in TGF-β expression in the BL/6-CD3FLAGmIR murine islets compared to BL/6 mice (Figure 4E).

### 3.2. Effect of IR Expressing T Cell Infiltration into Pancreatic Islets

**Figure 5:**
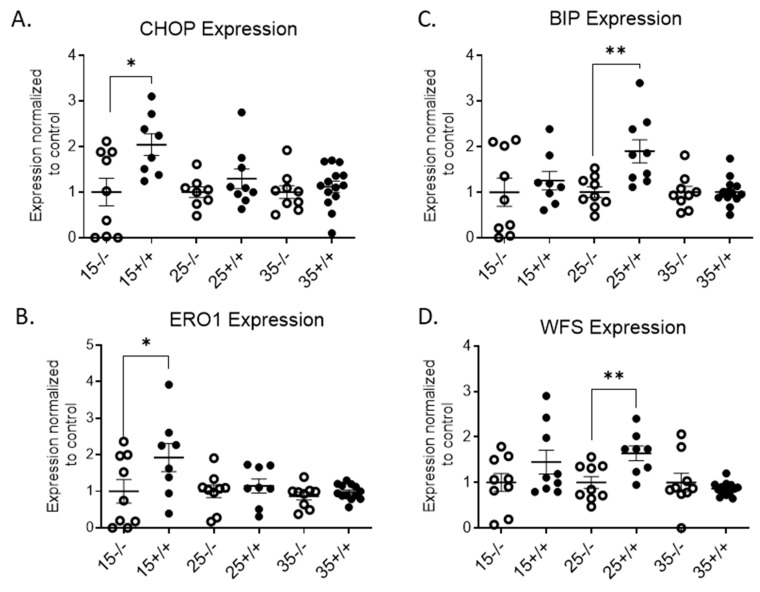
qRT-PCR analysis of BL/6 and BL/6-CD3FLAGmIR mice islet mRNA expression of CHOP, ERO1, BIP, and WFS. CHOP (**A**), ERO1(**B**), BIP (**C**), and WFS (**D**) islet mRNA expression at 15 (*n* = 3 mice with 3 replicates), 25 (*n* = 3 mice with 3 replicates) and 35 weeks (*n* = 4 mice with 3 replicates) of age. ^−/−^ indicates BL/6 mice and ^+/+^ indicates BL/6-CD3FLAGmIR mice. Both BL/6 and BL/6-CD3FLAGmIR results are compared to the control, beta-globin, a housekeeping gene. Unpaired student *t* test: * *p* ≤ 0.05, ** *p* ≤ 0.01.

Even though the transgenic mice did not become hyperglycemic [24], insulitis may still have had an impact on the islets. mRNA expression of endoplasmic reticulum (ER) stress markers in the transgenic mice islets was evaluated due to the IR^+^ T cell infiltration into the islets. ER stress markers such as CCAAT/enhancer-binding protein (C/EBP) homologous protein (CHOP), and ER oxidoreductin 1α (ERo1a) were significantly increased in the 15-week-old BL/6-CD3FLAGmIR mice islets (Figure 5A,B). Other ER markers such as Wolfram syndrome gene 1 (WFS-1), and binding immunoglobulin protein (BIP) expression were higher in the 25-week-old BL/6-CD3FLAGmIR mouse islets compared to those from BL/6 mice (Figure 5C,D). However, there were no changes observed in the ER stress marker expressions at 35 weeks of age.

**Figure 6:**
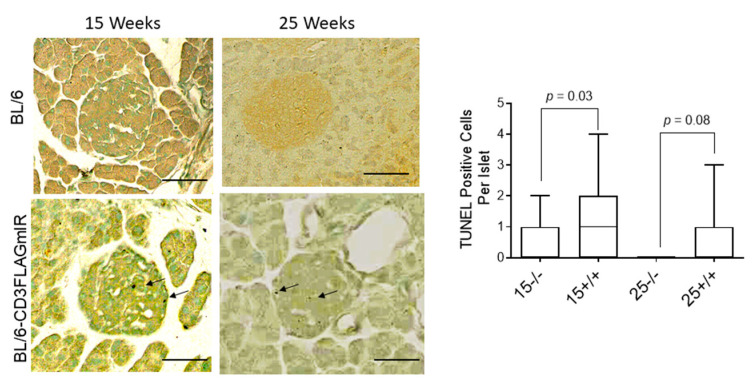
Tunnel staining and analysis. A. Immunohistochemistry labeling of pancreatic tissue sections from 15-week-old mice representing TUNEL^+^ apoptosis in the islet of the transgenic mice. Scale bar 50 µm. B. Quantitative analysis of total number of TUNEL-positive cells per islet based on immunohistochemistry TUNEL labelling of 15 (*n* = 3), and 25 week (*n* = 3) old mice pancreatic tissues. ^−/−^ indicates BL/6 mice and ^+/+^ indicates BL/6-CD3FLAGmIR mice. (*n* = 23 islets assessed per mouse).

Due to the presence of IR^+^ T cells and increased ER stress markers in the 15- and 25-week-old BL/6-CD3FLAGmIR mouse islets, we labeled the islets for the apoptosis marker, TUNEL, to determine if the inflammation and ER stress in the islet was causing beta cell death. We observed a significant number of TUNEL^+^ cells in the BL/6-CD3FLAGmIR mouse islets at 15 weeks of age compared to BL/6 mouse islets (Figure 6).

**Figure 7:**
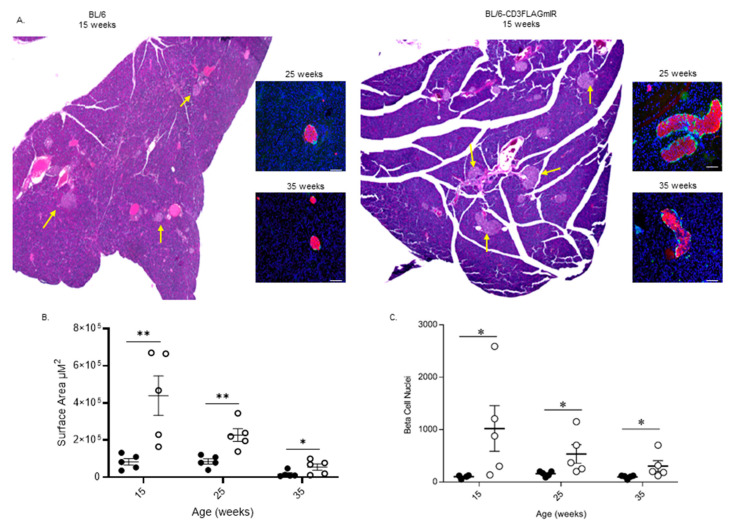
Analysis of islet area showing significantly increased islet area in BL/6-CD3FLAGmIR transgenic mice when compared to age-matched BL/6 mice. (**A**) An example image of whole H&E pancreatic tissue sections of 15-week-old BL/6 and BL/6-CD3FLAGmIR mice, 4×. Yellow arrows denote islets. Inset shows islets from 25 and 35-week-old BL/6 and BL/6-CD3FLAGmIR mice stained for insulin (red), glucagon (green), and DAPI for nuclei (blue). Insets scale bars are 100 µm (white line), pancreatic sections are represented to demonstrate the variation in islet size throughout the pancreas (slide scanned images at 4×) (**B**) Quantification of islet area from 10 islets from five separate animals BL/6 (black circles) or BL/6-CD3FLAGmIR transgenic (white circles) per group using ImageJ software. Each dot represents individual mice. Mann–Whitney test, * *p* ≤ 0.05, ** *p* ≤ 0.01. (**C**) Pancreatic islets analyzed for beta-cell nuclei counts from 10 islets from five separate animals BL/6 (black circles) or BL/6-CD3FLAGmIR transgenic (white circles) per group. Each dot represents an individual mouse. Mann–Whitney test, * *p* ≤ 0.05.

Upon H&E staining of pancreas tissue sections, the islets in the BL/6-CD3FLAGmIR mice appeared larger (Figure 7A). ImageJ analysis of islets confirmed a significant increase in surface area of the transgenic islets compared to age-matched BL/6 mice (Figure 7B). The increase in surface area in the BL/6-CD3FLAGmIR mice was due to the significant increase in beta-cells based upon the number of nuclei counted in insulin-stained islets (Figure 7C).

**Figure 8:**
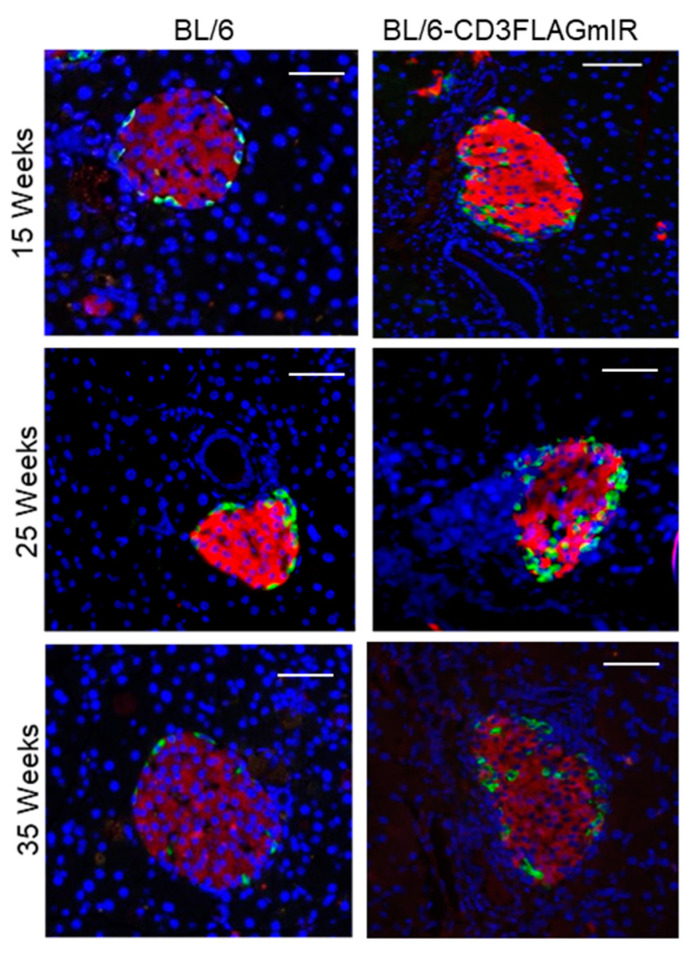
Islet morphology analysis by insulin (red) and glucagon (green). Immunofluorescence staining at 15, 25, and 35 weeks of age in BL/6 mice and BL/6-CD3FLAGmIR transgenic mice. (*n* = 3 mice per age group). Scale bar: 300 µm.

Along with islet size abnormality, islet cell disorganization was also noted by IF staining with anti-glucagon and anti-insulin to identify alpha and beta-cells, respectively (Figure 8). While BL/6 islets had the typical mantle conformation, alpha cells were found within the beta-cell cores starting at 15 weeks of age in the BL/6-CD3FLAGmIR mice (Figure 8).

**Figure 9:**
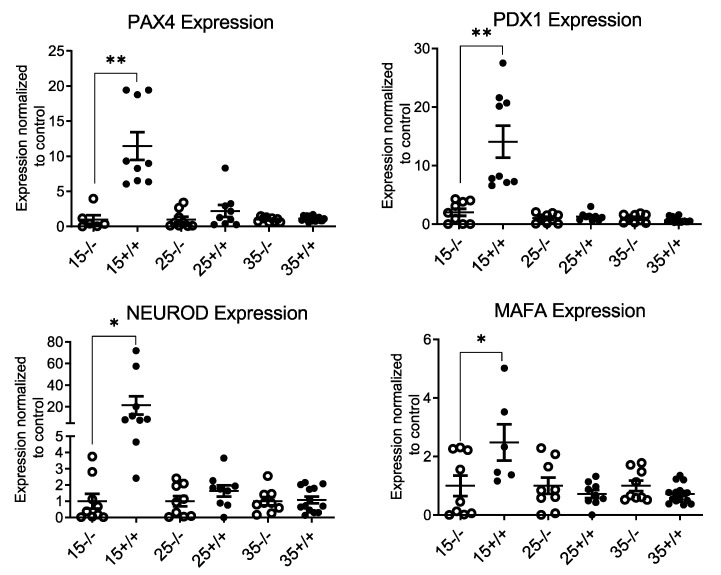
qRT-PCR analysis of BL/6 and BL/6-CD3FLAGmIR mice islet mRNA expression of PAX4, PDX1, NEUROD and MAFA. PAX4 (upper left), PDX1 (upper right), NEUROD (lower left) and MAFA (lower right) islet mRNA expression at 15 (*n* = 3 mice with 3 replicates), 25 (*n* = 3 mice with 3 replicates) and 35 weeks (*n* = 4 mice with 3 replicates) of age. ^−/−^ indicates BL/6 mice and ^+/+^ indicates BL/6-CD3FLAGmIR mice. BL/6 and BL/6-CD3FLAGmIR responses are compared to the control, which is the beta-globin housekeeping gene. Unpaired student *t* test: * *p* ≤ 0.05, ** *p* ≤ 0.01.

IR expressing T cell infiltration into the transgenic mice islet may promote beta cell proliferation to compensate for the inflammation and beta cell loss. Therefore, mRNA expression of pancreatic and duodenal homeobox 1 (Pdx-1), V-maf musculoaponeurotic fibrosarcoma oncogene homolog A (MAFA), Neurogenic differentiation (NeuroD), and paired box 4 (Pax4) was evaluated to determine the effect of IR^+^ T cell infiltration into the islet on beta cell differentiation and maintenance. mRNA expression of beta cell differentiation and maintenance markers was significantly higher in BL/6-CD3FLAGmIR mice islets at 15 weeks of age with no changes in the islets of 25- and 35-week-old BL/6-CD3FLAGmIR mice (Figure 9).

**Figure 10:**
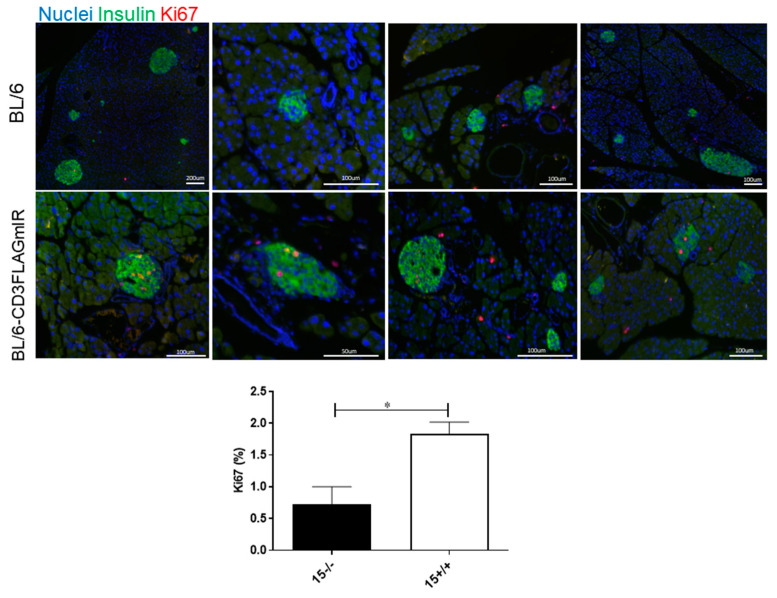
Immunofluorescence labelling of pancreatic tissue sections from 15-week-old mice representing Insulin^+^Ki67^+^ beta cells in the islet of the transgenic mice. Quantitative analysis indicates the percentage of Ki67^+^ cells based on Ki67 labelling of 15-week-old BL/6 and BL/6-CD3FLAGmIR pancreatic tissue (*n* = 3 mice, *n* = 23 islets analyzed per mouse). ^−/−^ indicates BL/6 mice and ^+/+^ indicates BL/6-CD3FLAGmIR mice. Unpaired student t test: * *p* ≤ 0.05.

To investigate the implications of increased beta cell differentiation and maintenance markers, we stained the 15-week-old BL/6-CD3FLAGmIR and Bl/6 wildtype pancreatic tissue sections with a proliferation marker, Ki67. The percentage of Ki67^+^ beta cells was significantly higher in the BL/6-CD3FLAGmIR mice specifically in the glucagon-stained islets (Figure 10).

**Figure 11:**
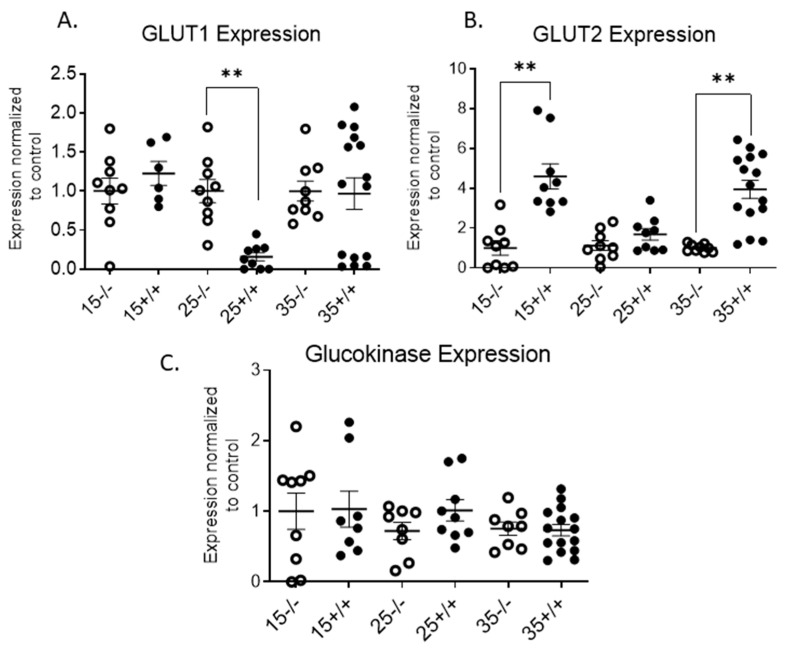
qRT-PCR analysis of BL/6 and BL/6-CD3FLAGmIR mice pancreatic islet mRNA expression of GLUT1, GLUT2, and Glucokinase. GLUT1 (**A**), GLUT2 (**B**), and Glucokinase (**C**) islet mRNA expression at 15 (*n* = 3 mice with 3 replicates), 25 (*n* = 3 mice with 3 replicates) and 35 weeks (*n* = 4 mice with 3 replicates) of age. ^−/−^ indicates BL/6 mice and ^+/+^ indicates BL/6-CD3FLAGmIR mice. Both BL/6 and BL/6-CD3FLAGmIR results are compared to the control, beta-globin, a housekeeping gene. Unpaired student *t* test: ** *p* ≤ 0.01.

Islet beta cells express GLUT2 on their surface for glucose transport. GLUT2 expression was significantly increased at 15 and 35 weeks of age (Figure 11A,B). Due to the changes in GLUT2 expression on the transgenic mice beta cells, we also evaluated the glucokinase expression in the pancreatic islet. Glucokinase has its highest expression in pancreatic beta cells and the liver [28]. Glucokinase acts as a glucose sensor for the beta cell and controls the rate of glucose entry into the glycolytic pathway (glucose phosphorylation) [28]. Glucokinase mRNA expression in the transgenic mouse islets was normal, similar to BL/6 mice (Figure 11C). Overall, changes in the islet ER stress markers, beta cell differentiation and maintenance expressions indicate that IR^+^ T cell infiltration into the pancreas affects islet cells.

### 3.3. BL/6-CD3FLAGmIR Transgenic Animal Metabolic Abnormalities

**Figure 12:**
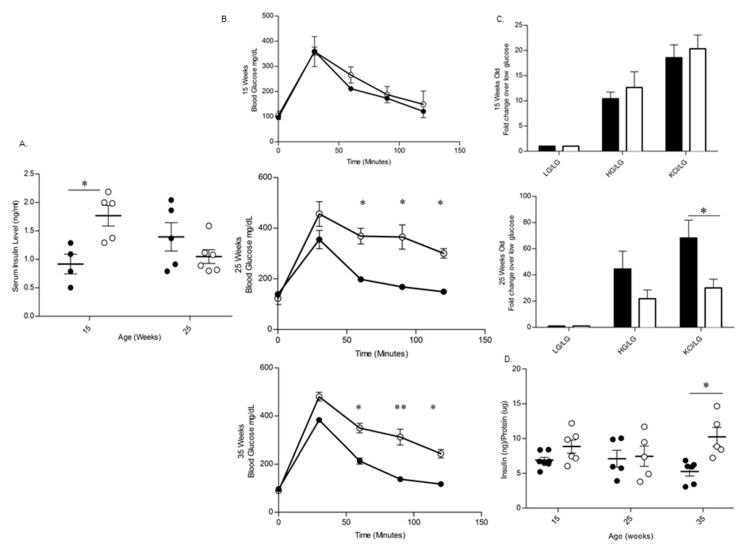
Metabolic study of BL/6 and BL/6-CD3FLAGmIR transgenic mice. (**A**) Non-fasting serum insulin levels of BL/6 (black circles) and BL/6-CD3FLAGmIR transgenic male mice (white circles). Each dot represents an individual mouse. One-way ANOVA followed by Tukey’s multiple comparison test, * *p* ≤ 0.05. (**B**) Intraperitoneal glucose tolerance test comparing BL/6 (black circles) and BL/6-CD3FLAGmIR transgenic male mice (white circles) at 15, 25, and 35 weeks of age (n = 4 mice per group). Unpaired student *t* test, * *p* ≤ 0.05, ** *p* ≤ 0.01. (**C**) Glucose stimulated insulin secretion in vitro at age 15 (*n* = 4) and 25 weeks (*n* = 5) in BL/6 (Black bars) and BL/6-CD3FLAGmIR (White bars). Welch’s *t* test, * *p* ≤ 0.05. (**D**) Total Insulin Content of pancreas from BL/6 (black circles) and BL/6-CD3FLAGmIR transgenic male mice (white circles) at 15, 25, and 35 weeks of age. Each dot represents an individual mouse, Two-way ANOVA followed by Tukey’s multiple comparison test, * *p* ≤ 0.05.

To understand the functional implications of islet morphological changes and the infiltration of the FLAG-tagged IR^+^ T cells into the islets, in vivo metabolic profiles were also examined. Non-fasting serum insulin levels were significantly higher in BL/6-CD3FLAGmIR at 15 weeks of age than age-matched BL/6, whereas this difference was no longer detected at 25 weeks of age (Figure 12A). Intraperitoneal glucose tolerance test (IPGTT) was performed on BL/6-CD3FLAGmIR and BL/6 mice at 15, 25, and 35 weeks of age (Figure 11B). BL/6-CD3FLAGmIR mice were found to have impaired glucose tolerance compared to BL/6 mice at 25 and 35 weeks of age (Figure 12B). In order to determine the cause of the impaired glucose tolerance seen in vivo, islets from BL/6-CD3FLAGmIR and BL/6 mice were isolated and examined for their insulin secretory capacity in vitro. Insulin secretion was comparable in islets obtained from 15-week-old BL/6 and BL/6-CD3FLAGmIR mice upon stimulation with high glucose (22 mM) and potassium chloride (KCl) treatment. In contrast, there was a decrease in insulin secretion at 25 weeks of age upon stimulation with high glucose (22 mM) and a significant decrease with KCl treatment (Figure 12C). There were no differences in the total pancreatic insulin content of BL/6-CD3FLAGmIR compared to the BL/6 mice at 15 and 25 weeks of age (Figure 12D). Although improved insulin content was reported at 35 weeks of age in BL/6-CD3FLAGmIR mice, abnormal glucose tolerance was still observed.

### 3.4. Adoptive Transfer Studies

**Figure 13:**
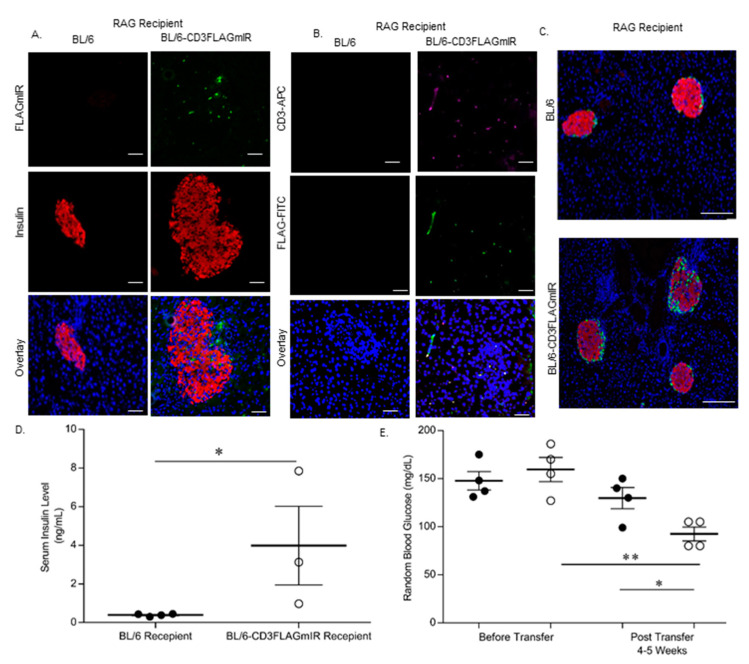
Adoptive transfer of whole splenocytes from either BL/6-CD3FLAGmIR mice, BL/6 mice, or vehicle PBS into C57BL/6 RAG^−/−^ mice. (**A**) Immunofluorescence staining of RAG^−/−^ recipient mice islets stained for insulin (Red), FLAG expressing T cells (Green), and DAPI for nuclei (Blue). Scale bar: 100 µm. (**B**) Immunofluorescence staining of pancreatic tissue stained with anti-CD3 (magenta) and anti-FLAG (Green) to demonstrate the presence of FLAGmIR^+^ T cells in the recipient mice islets. Scale bar: 100 µm. (**C**) Immunofluorescence staining of BL/6 and BL/6-CD3FLAGmIR recipient islets for insulin (Red) and glucagon (green). Scale bar: 300 µm. (**D**) Non-fasting serum insulin levels of recipient RAG^−/−^ mice at 5 weeks post-transfer of either BL/6 splenocytes (black circle), or BL/6-CD3FLAGmIR splenocytes (white circle). Each dot represents an individual mouse. Mann–Whitney test, * *p* ≤ 0.05. (**E**) Random blood glucose levels from recipient mice before and post-transfer with BL/6 splenocytes (black circle) or BL/6-CD3FLAGmIR transgenic splenocytes (white circle). Each dot represents an individual mouse. One-way ANOVA followed by Tukey’s multiple comparison test * *p* ≤ 0.05, ** *p* ≤ 0.01.

Adoptive transfer was completed to ascertain whether the transgenic phenotype could be transferred to an immune deficient RAG^−/−^ model. C57BL/6 RAG^−/−^ recipient mice receiving BL/6 splenocytes had normal islet size and architecture without infiltrating cells, whereas C57BL6 RAG^−/−^ recipient mice that received BL/6-CD3FLAGmIR splenocytes had increased islet size and disrupted architecture with infiltrating FLAG-tagged IR^+^ CD3^+^ T cells. Thus, the phenotype observed in the transgenic mice could be transferred to the RAG^−/−^ recipient mice by 4–5 weeks post-transfer of transgenic splenocytes (Figure 13A–C). Interestingly, there was a significant increase in the insulin levels in the blood serum of the animals that received BL/6-CD3FLAGmIR splenocytes 5 weeks post-transfer, and a concomitant decrease in random blood glucose levels when compared to animals that received BL/6 splenocytes (Figure 13D,E).

**Figure 14:**
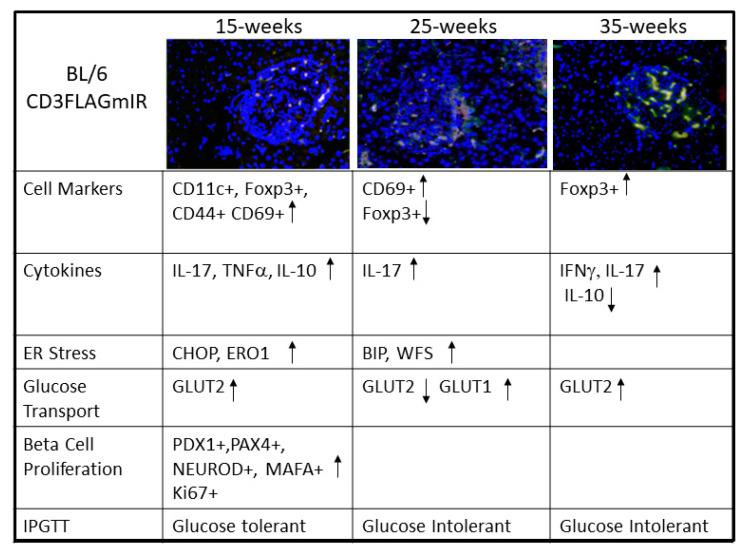
Summary of manuscript findings, demonstrating the effects of CD3FLAGmIR T cells on cell markers, cytokines, ER stress, glucose transport, beta cell proliferation, and glucose tolerance over time.

Figure 14 summarizes the findings of the paper and is discussed in the discussion section as a model of what happens when non-antigen-specific IR^+^ T cells migrate into islets and the consequences for the islets.

## 4. Discussion

In this study, we demonstrated that IR^+^ T cell infiltration into the islets causes morphological changes and metabolic disturbances in the BL/6-CD3FLAGmIR transgenic mouse islets. Although alteration in the beta cell functionality was observed in BL/6-CD3FLAGmIR mice, the blood sugar levels in these mice did not rise to the level of overt diabetes [24]. Recently, multiple studies have reported that T cell trafficking to the pancreatic islet is not necessarily antigen-specific [17,29,30]. Therefore, although IR^+^ T cell infiltration into the islet of BL/6-CD3FLAGmIR mice did not cause hyperglycemia, invading IR^+^ T cells had a metabolic impact on the beta cells. This model provides novel insights to the development of insulitis and the impact of insulitis on islet cells.

Sandor A. M. et al. reported that the islet vascular barrier is highly restrictive to the T cell entry and CD11c^+^ cells are necessary for effective entry of lymphocytes into the previously inflamed NOD islets [17]. In BL/6-CD3FLAGmIR mice, we observed an increased expression of CD11c^+^ in 15-week-old islets that may be indicative of these cells assisting IR^+^ T cells to cross the islet vascular barrier initially and enter the islet. The increase of CD11c+ expression was not found at 25 or 35 weeks of age, implicating this as an initial mechanism, but not sustained. Insulitis significantly increased in the 25 and 35-week-old BL/6-CD3FLAGmIR mice. Moreover, inflammatory IFNγ and IL-17 expression were significantly increased in the 35-week-old transgenic mouse islets with a significantly lowered IL-10 expression. This indicates that islet inflammation increased with age in the transgenic mice with potentially decreased immunosuppression. There were no changes in the CD68 expression (a marker for circulating and tissue resident macrophages) in BL/6-CD3FLAGmIR mice islets indicating that insulitis was not conferred due to macrophage infiltration.

Interestingly BL/6-CD3FLAGmIR murine islets show a few characteristics observed in the NOD T1D murine model and there are similarities with human diabetes. For example, the majority of FLAG-tagged mIR^+^ T cells in the transgenic homozygous BL/6-CD3FLAGmIR mice were CD8^+^ T cells [24]. Interestingly, the majority of T cells in human insulitis are CD8^+^ T cells [31]. The BL/6-CD3FLAGmIR transgenic mouse islets had high expression of the activation/adhesion molecules such as CD44^+^, CD69^+^, and CD62L^-^. CD44 expression on T lymphocytes has been shown to assist in T cell trafficking, in particular, into sites of inflammation [32,33,34]. Our studies indicate the splenocytes are CD44^+^ and CD62L^+^ but, when the CD3^+^ T cells get to the islets, the CD62L expression is no longer significant. These cells now exhibit an activated phenotype with only CD44^+^ and CD69^+^ activation markers. This suggests that CD3^+^ cells may utilize the CD62L^+^ to get into the islet, but once there, it is no longer highly expressed [33,35,36]. The CD44^+^, CD62L^-^ and CD69^+^ markers that were observed in the BL/6-CD3FLAGmIR mice were also observed in CD8^+^ T cells in NOD mice [37]. The appearance of such cells in prediabetic NOD mice affected their glucose tolerance without causing hyperglycemia, similar to the metabolic changes observed in the BL/6-CD3FLAGmIR transgenic mice [24,35].

In addition, we observed a significant increased expression of FOXP3^+^ T regulatory cell and IL-10 in the BL/6-CD3FLAGmIR mouse islets at 15 weeks of age. T regulatory cell activity in the transgenic mouse islets at 15 weeks of age may be to suppress the inflammation caused by infiltrating IR^+^ T cells. In human, even a moderate regulatory response can be enough to prevent development of clinical T1D in individuals genetically predisposed to T1D [38]. IL-10 mediated immunosuppression could be a reason for normal glucose tolerance and glucose-stimulated insulin secretion in the 15-week-old BL/6-CD3FLAGmIR transgenic mice. IL-17 is secreted by activated T cells and is a pro-inflammatory cytokine [39]. Activated CD44^+^ T cells were significantly present at 15 weeks and CD69^+^ T cells were significantly present at 15 weeks and 25 weeks. Pro-inflammatory cytokines can induce beta cell ER stress in vitro and can contribute to beta cell loss in T1D [40,41]. We observed increased expression of ER stress markers in the 15 and 25-week-old transgenic mice islets. CHOP, and ERO1 expression was higher in the 15-week-old transgenic mice islets while BIP, and WFS expression was significantly increased in the 25-week-old transgenic mice islets. Similar heterogeneity of ER stress markers in the islets was also observed in patients with T1D [41]. In addition, we observed a significantly increased level of apoptosis in the islets of BL/6-CD3FLAGmIR mice at 15 weeks of age. This could be a result of the inflammation caused by infiltrating IR^+^ T cells and the increased ER stress in the transgenic mouse islets.

Prediabetic NOD mice have a larger islet area than other strains of mice including C57BL/6 mice and BALB/c [42]. Furthermore, prediabetic NOD mice have increased islet area and beta-cell replication compared to NOD.scid mice [43]. Increased islet size is also observed in human TID, where T1D organ donors that had insulitis had a significantly higher insulin-positive beta cell area compared to T1D organ donors without insulitis [19]. The increase in beta-cell area is thought to be due to inflammatory or metabolic stress [43,44,45]. The BL/6-CD3FLAGmIR transgenic mice had an increased islet size and higher number of beta-cell nuclei as compared to BL/6 mice from 15 to 35 weeks of age. We also observed increased expression of Pdx1, Pax4, MafA, and NeuroD in the 15-week-old BL/6-CD3FLAGmIR transgenic mice islets. The overexpression of Pdx1, Pax4, and NeuroD has been shown to promote embryonic stem cell differentiation into insulin-producing beta cells [46]. The increased percentage of insulin-producing Ki67^+^ cells at 15 weeks of age in the BL/6-CD3FLAGmIR transgenic mice correlated closely with the overexpression of Pdx1, Pax4, and NeuroD in the islets. This indicates IR^+^ T cell infiltration into the islet, and increased ER stress within the islet may have stimulated beta cell proliferation at 15 weeks of age. At 25 and 35 weeks of age, insulitis was increased, but the beta cell differentiation and maintenance expression were not observed significantly in the islet, indicating beta cells might not be able to compensate for the inflammation caused by infiltrating IR^+^ T cells. This closely resembles beta cell proliferation in human T1D [47]. Willcox A. et al. reported increased islet cell proliferation in patients with recent T1D onset while patients with a long duration of diabetes do not display islet cell proliferation [47]. Along with beta-cell area differences, islet morphology changed in the transgenic mice. Normally in murine islets, glucagon secreting cells are found along the mantle, while insulin secreting beta-cells are at the core [48]. By 15 weeks of age the transgenic mice displayed disrupted islets containing glucagon secreting cells within the beta cell core. Increased islet mass and disorganized islets have also been observed in a C57BL/6 transgenic mouse model of type 2 diabetes (T2D) [48].

The BL/6-CD3FLAGmIR transgenic mice did not develop elevated fasted or random blood glucose levels [24]. Although BL/6-CD3FLAGmIR mice had increased islet area, beta cell nuclei, and serum insulin levels at 15 weeks of age, there was no significant difference in islet insulin content, in vitro GSIS, or in vivo glucose tolerance at this point. At 15 weeks of age, insulitis, caused by the IR^+^ T cell infiltration into the transgenic mice islet, may be compensated for by the expansion of beta cell area, increased serum insulin levels and GLUT2 expression [49]. In GLUT2-null mice, the re-expression of GLUT1 or GLUT2 restores normal glucose stimulated insulin secretion and glucose tolerance [49]. In contrast, by 25 weeks, BL/6-CD3FLAGmIR mice became glucose intolerant on in vivo testing, and this impairment is likely explained by the subnormal islet insulin secretion seen in vitro upon stimulation with the secretagogue KCl. This indicates that increased infiltration of IR^+^ T cells into pancreatic islets with age can ultimately cause impairment in beta cell function. In the prediabetic condition of both T1D and T2D, postprandial hyperglycemia may be the first blood glucose abnormality observed [50,51]. The blood glucose patterns in this model may most closely resemble these prediabetic states.

In order to confirm the morphological abnormalities in BL/6-CD3FLAGmIR mice are due to FLAG-tagged mIR^+^ T cells, we performed adoptive transfer of splenocytes from BL/6-CD3FLAGmIR mice into immunodeficient C57BL/6RAG^−/−^ mice. The transfer model mimicked the transgenic mouse in a much-abbreviated time frame with all of the morphological abnormalities visible in the transfer mice noted at 5 weeks that were found in BL/6-CD3FLAGmIR mice by 15 weeks of age. This is reminiscent of the transfer of spleen cells from diabetic NOD mice to nondiabetic irradiated NOD or NOD-SCID mice, not for the transfer of diabetes, but for the transfer of T cell insulitis into the pancreas [6]. Furthermore, the transfer of transgenic splenocytes into the C57BL/6RAG^−/−^ mice lowered random blood glucose levels significantly, in opposition to what occurs in diabetes. Thus, we have shown that that IR+ T cells can respond to insulin by migration in vitro [24] and in vivo [24] and now by cell transfer in vivo. This new model of non-antigen-specific T cell invasion of the pancreas is due to the expression of IRs on the T cell surface and chemotaxis to insulin.

The following model is based on Figure 14 and other findings in the paper. Along with IR expression on the transgenic T cells, the cells are CD44^+^ and CD62L^+^, giving them an adhesion molecule to enter the pancreas and the CD62L can bind to selectin on the vascular cell walls [32,33,34,35,36]. CD11c cells, as evidenced by qPCR, are in place in the transgenic islets to assist the T cell entrance into the islet [17]. Once in the islet at 15 weeks the T cell invasion causes ER stress in the islet and beta cell proliferation as shown by increased islet size, increased number of beta cell nuclei, increased PAX1, PAX-4, and NEUROD and Ki 67^+^staining. There is also some apoptosis as shown by the TUNNEL staining. Although there is significant expression of IL-17 and TNFα at 15 weeks, the Foxp3+ cells are up and IL-10 is up and insulin in the serum is up, leading to a normal glucose stimulated insulin release in vivo and in vitro. At 25 weeks, the CD69^+^ cells are still significantly up. Also, the IL-17 is significantly increased and IL-10 is not significantly different compared to BL/6 mice islets, and further ER stress markers are up (BIP, WFS). Insulin in the serum is not significantly different from BL/6 mice. Stimulation by the insulin secretagogue KCL in vitro is abnormal in the 25-week-old mice, and IPGTT, in vivo, showed the transgenic mice to be glucose intolerant. At 35 weeks in the transgenic mice, although Foxp3+ cells are in evidence, IL-10 is significantly down, while proinflammatory IL-17 and IFNγ are significantly increased with a continued IPGTT that is abnormal in vivo. Therefore, even non-antigen-specific insulitis by IR^+^T cells impacts islet cells and insulin secretion.

## Figures and Tables

**Table 1:** Reaction conditions used for qRT-PCR analysis.

Reagent	20 µL Reaction Volume	70 µL Reaction Volume
2× SYBR Green Master Mix	10 µL	35 µL
Forward Primer	1 µL	3.5 µL
Reverse Primer	1 µL	3.5 µL
DI Water	7 µL	24.5 µL
cDNA (150 ng–200 ng/µL)	1 µL	3.5 µL

**Table 2:** Murine primers (5′–3′) used for qRT-PCR studies.

FOR_WFS	CCATCAACATGCTCCCGTTC
REV_WFS	GGGTAGGCCTCGCCATACA
FOR_BIP	TTCAGCCAATTATCAGCAAACTCT
REV_BIP	TTTTCTGATGTATCCTCTTCACCAGT
FOR_ERO1	GCTCTCTCCAAAGTGCTTCCA
REV_ERO1	TGCATCCTGAACTTTATTCCCA
FOR_PDX1	GACACATCAAAATCTGGTTCCAAA
REV_PDX1	TCCCGCTACTACGTTTCTTATCTTC
FOR_GAPDH	AAGGTCATCCCAGAGCTGAA
REV_GAPDH	ATGTAGGCCATGAGGTCCAC
FOR_FLAGmIR	CCATGTCTGCACTTCTGACTAGCTCTT
REV_FLAGmIR	CCATAGACACGGAAGAGAGCAGGTAATC
FOR_IFNG	CGGCACAGTCATTGAAAGCCTA
REV_IFNG	GTTGCTGATGGCCTGATTGTC
FOR_IL-17	GCTCCAGAAGGCCCTCAGA
REV_IL-17	CTTTCCCTCCGCATTGACA
FOR_IL-10	CACAAAGCAGCCTTGCAGAA
REV_IL-10	AGAGCAGGCAGCATAGCAGTG
FOR_TNFA	GATCTCAAAGACAACCAACATGTG
REV_TNFA	CTCCAGCTGGAAGACTCCTCCCAG
FOR_TGFB	CACCGGAGAGCCCTGGATA
REV_TGFB	TGTACAGCTGCCGCACACA
FOR_Beta-Globin	CCAATCTGCTCACACAGGATA
Rev_Beta-Globin	CCTTGAGGCTGTCCAAGTGAT
FOR_WFS	CCATCAACATGCTCCCGTTC
REV_WFS	GGGTAGGCCTCGCCATACA
FOR_BIP	TTCAGCCAATTATCAGCAAACTCT
REV_BIP	TTTTCTGATGTATCCTCTTCACCAGT
FOR_ERO1	GCTCTCTCCAAAGTGCTTCCA
REV_ERO1	TGCATCCTGAACTTTATTCCCA
FOR_PDX1	GACACATCAAAATCTGGTTCCAAA
REV_PDX1	TCCCGCTACTACGTTTCTTATCTTC
FOR_PAX4	GTGTTGGCTCCAGTTCTTCC	
REV_PAX4	AACCAAACCCTCACCGTGTC	
FOR_GLUT2	GGCTAATTTCAGGACTGGTT	
REV_GLUT2	TTTCTTTGCCCTGACTTCCT	
FOR_MAFA	TTCAGCAAGGAGGAGGTCAT	
REV_MAFA	CCGCCAACTTCTCGTATTTC	
FOR_GLUT1	CATCCTTATTGCCCAGGTGTTT	
REV_GLUT1	GAAGACGACACTGAGCAGCAGA	
FOR_NEUROD1	TATTGCGTTGCCTTAGCACT	
REV_NEUROD1	CATCCTCTTGAGTGTTATGG	
FOR_CD11C	ACACAGTGTGCTCCAGTATGA	
REV_CD11C	GCCCAGGGATATGTTGACAGC	
FOR_CD68	CTTCCCACAGGCAGCACAG	
REV_CD68	AATGATGAGAGGCAGCAAGAGG	
FOR_FOXP3	ACTCGCATGTTCGCCTACTTCAG	
REV_FOXP3	GGCGGATGGCATTCTTCCAGGT	
FOR_CD44	TGAAACATGCAGGTATGGGT	
REV_CD44	GCTGAGGCATTGAAGCAATA	
FOR_CD69	TGGTCCTCATCACGTCCTTAATAA	
REV_CD69	TCCAACTTCTCGTACAAGCCTG	
FOR_CD62L	CATTCCTGTAGCCGTCATGG	
REV_CD62L	AGGAGGAGCTGTTGGTCATG	
FOR_GLUCOKINASE	GAATCTTCTGTTCCACGGAG	
REV_GLUCOKINASE	AGTGCTCAGGATGTTAAGGA	
FOR_CHOP	CCACCACACCTGAAAGCAGAA	
REV_CHOP	AGGTGAAAGGCAGGGACTCA	
FOR_PAX4	GTGTTGGCTCCAGTTCTTCC	
REV_PAX4	AACCAAACCCTCACCGTGTC	
FOR_GLUT2	GGCTAATTTCAGGACTGGTT	
REV_GLUT2	TTTCTTTGCCCTGACTTCCT	

**Table 3:** Experimental protocol used for qRT-PCR analysis.

Step	Temperature (°C)	Time
1. Initial Denaturation	95	3 min
2. Template Denaturation	95	30 s
3. Primer Annealing	Depending upon the Forward and Reverse Primer melting temperature	30 s
4. Primer Extension	72	30 s
5. Cycle Repeat (steps 2–4) 39 times’		
6. Plate Read for SYBR Green after every cycle		

## Data Availability

Not applicable.
